# Wide Dynamic Range CMOS Potentiostat for Amperometric Chemical Sensor

**DOI:** 10.3390/s100301782

**Published:** 2010-03-04

**Authors:** Wei-Song Wang, Wei-Ting Kuo, Hong-Yi Huang, Ching-Hsing Luo

**Affiliations:** 1 Department of Electrical Engineering, National Cheng Kung University, Tainan, Taiwan; E-Mails: n2895145@ccmail.ncku.edu.tw (W.-S.W.); superman1120@gmail.com (W.-T.K.); robin@ee.ncku.edu.tw (C.-H.L.); 2 Graduate Institute of Electrical Engineering, National Taipei University, No. 151, University Road, San Shia, Taipei 237, Taiwan

**Keywords:** potentiostat, dynamic range, amperometric sensor, fully differential, transimpedance

## Abstract

Presented is a single-ended potentiostat topology with a new interface connection between sensor electrodes and potentiostat circuit to avoid deviation of cell voltage and linearly convert the cell current into voltage signal. Additionally, due to the increased harmonic distortion quantity when detecting low-level sensor current, the performance of potentiostat linearity which causes the detectable current and dynamic range to be limited is relatively decreased. Thus, to alleviate these irregularities, a fully-differential potentiostat is designed with a wide output voltage swing compared to single-ended potentiostat. Two proposed potentiostats were implemented using TSMC 0.18-μm CMOS process for biomedical application. Measurement results show that the fully differential potentiostat performs relatively better in terms of linearity when measuring current from 500 pA to 10 uA. Besides, the dynamic range value can reach a value of 86 dB.

## 1. Introduction

The field of electrochemical study often involves sensor devices which are used to measure certain quantity of analytes within a given solution. In the event where the sensor is detecting some specific analytes in a solution, a corresponding output signal is subsequently generated. Most often, the output signal takes the form of potential (*i.e.*, voltage) and current. Additionally, this output signal is directly proportional to the concentration quantity of the analytes.

One of the common methods of detecting analytes is through the used of amperometric sensor, in which it utilizes a potentiostat hardware, which is used to control the electrode cells for running electroanalytical experiments. Generally, the structure of the potentiostat hardware utilized in amperometric sensor contains three kinds of electrode cells. The first electrode cell is called a working electrode (WE). Its function is to serve as a platform on which the electrochemical reaction takes place. The second is referred to as reference electrode (RE), and its function is to measure any potential quantity present in WE electrode. The voltage at the reference electrode of a potentiostat can be affected by the current that goes through this terminal. It is usually not allowed to have current go though the reference electrode. A high input impedance is suggested to maintain this requirement which can be easily implemented by connecting the reference electrode to the gate terminal of a MOSFET. Furthermore, integrating the potentiostat into CMOS chip may reduce the system cost and hardware size. It is even more important for a system-on-chip when this function has to be combined with some other applications. Lastly, the third electrode is called counter electrode that serves as a conductor which supplies current required for electrochemical reaction at WE electrode.

**Figure 1.**
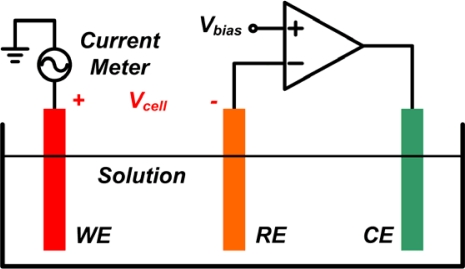
Conceptual drawing of three electrode amperometric electrochemical sensor and potentiostat.

Figure 1 shows the basic conceptual structure of an amperometric sensor with three-electrode potentiostat device. The potential difference, Vcell, between WE and RE electrodes is measured by a potentiostat and is kept at certain potential value for specific function. This is accomplished by sinking or sourcing currents from or into the sensor through electrode CE, which in turn measures the current.

The fundamental operation of each function can be realized through two different circuit configurations: potential control and current control.

### 1.1. Potential Control Configuration

In this configuration, the value of the cell potential can be controlled in three different methods: grounded counter electrode [1], grounded working electrode [2,3] and virtual grounded working electrode [4]. Even though the virtual grounded working electrode configuration can prevent current from flowing into RE, the working electrode has no direct connection to the true ground. Any connection present in the potentiostat that is not prudently shielded may cause it to pick some environmental noise and interference which may produce significant noise levels at the output of the transimpedance amplifier. The output connection between control amplifier and counter electrode produces a very large capacitive value that could contribute instability factor on the control amplifier. This situation goes the same for grounded counter electrode. Furthermore, the grounded counter electrode configuration has evidently seen to have more complex situation and requires more components, making it more vulnerable to common interference and is often prone to component mismatches.

### 1.2. Current Measurement Configuration

There are, in general, two configurations being used to measure the amount of cell current: readout current through working electrode and readout current through counter electrode. In the first configuration, literatures [1], [4] and [5] have employed transimpedance amplifier to read out current from WE. The read-out current configuration offers a number of advantages over the second configuration: it is easier to realize and few currents are measured due to switching current measurement resistor to higher values. The disadvantages, however, include no direct connection between working electrode and true ground, which causes to acquire environmental noise and some form of interference that produces significant noise level at the output of transimpedance amplifier, and instability and oscillation in the potential control loop due to the inductive behavior of input resistance present in the transimpedance amplifier. In addition, the series connection of transimpedance amplifier and electrochemical cell, which has very large capacitive components has greatly contributes to the inductivity of the amplifier [6]. On the other hand, literatures [7–13] utilized two-electrode sensor current conveyor, in which a working electrode is still held at virtual ground picking any presence of environmental noise and interference. However, the nonlinear property of the transistor resistance between drain and source terminals degrades the performance of linearity of the potentiostat. In the second configuration, the three-electrode current conveyor has been fully experimented with in [3] and [14], however, limitation on saturated voltage and nonlinearity issues have been encountered. But working electrode is held at a true ground preventing noise and interference. Moreover, literature [14] has investigated the method of current mirror conveyor as an alternative topology of three-electrode sensor. Unfortunately, the presence of current mirror mismatch has caused the potentiostat linearity to degrade.

To alleviate the issues on voltage saturation and linearity, this paper presents a single-ended potentiostat topology with a new interface connection between sensor electrodes and potentiostat. In addition, a large harmonic distortion may limit the detectable dynamic range when the current is too low. Therefore a fully-differential potentiostat that provides a wider output swing to perform better linearity is presented to improve the linearity. The additional fully-differential potentiostat rejects existing second-order harmonic distortions and eventually help improve circuit linearity. Section 2 presents the proposed single-ended potentiostat topology and fully-differential potentiostat topology along with the circuit realizations. In Section 3, the measurement results of the potentiostat circuits are presented. Finally, the conclusion of this paper is reported in Section 4.

## 2. Proposed Potentiostat Topology

### 2.1. Single-ended Potentiostat

**Figure 2.**
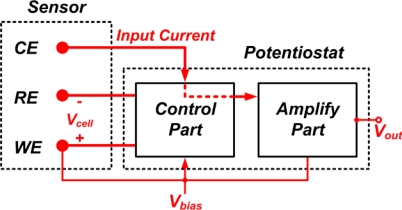
Block diagram of the single-ended potentiostat.

Figure 2 shows the block diagram of a single-ended potentiostat topology. It consists of two major blocks, control part and the amplifying part. These blocks determine the major function of the potentiostat system. The following is the description of these blocks.

#### a) Control Part

The control part maintains the cell voltage at desired potential level. It forces the voltage between WE and RE to be the same as voltage bias (V_bias_) by driving or sourcing current from or into counter electrode.

#### b) Amplifying Part

**Figure 3.**
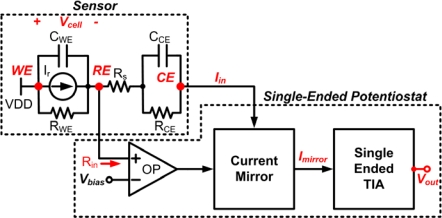
The building blocks of single-ended potentiostat.

The amplifying part reads out cell current from the counter electrode and converts such current signal into a voltage signal. Figure 3 shows the building blocks of a single-ended potentiostat with its new interface connection from sensor electrodes. The proposed configuration offers a number of advantages. First, the reference electrode is directly connected to the input of control amplifier, which can prevent the current from flowing into RE. Second, the output of control amplifier is connected to the gate terminal of mirror transistor instead of counter electrode directly. This can prevent the control amplifier from being loaded with large capacitance of the counter electrode. Finally, the connection between WE to V_DD_ can prevent from picking up environmental noise, which may cause to produce significant noise level at the output of the transimpedance amplifier. Furthermore, the variable voltage swing of the sensor cell has a wider range than the conventional structure. The bias voltage is applied to the sensor between the working and reference electrodes such that:

(1) $$V_{\mathrm{cell}}=V_{\mathrm{WE}}-V_{\mathrm{RE}}=V_{\mathrm{DD}}-V_{\mathrm{bias}1}$$

**Figure 4.**
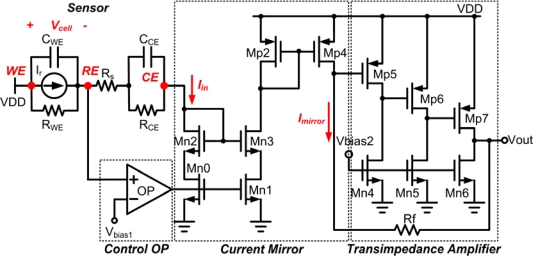
The transistor level of the single-ended potentiostat.

Figure 4 shows the proposed single-ended potentiostat which consists of three building blocks: control amplifier, current mirror, and single-ended transimpedance.

The design of current mirror in Figure 4 must consider two key points. First, there must be precise copying of current to the next stage; thus a cascode current mirror is adopted to attain good linearity. Second, the output of control amplifier must be connected to the gate of drain terminal topology [11] to reduce mismatch of current mirror, which is induced by the different drain-source voltage on mirror transistors, M_n2_ and M_n3_. Then it is transmitted through another current mirror composed of M_p2_ and M_p4_. The current mirror function can be expressed as follows:

(2) $$I_{\mathrm{out}}=\frac{\left(W/L\right)n3\cdot \left(W/L\right)p4}{\left(W/L\right)n2\cdot \left(W/L\right)p2}\cdot I_{\mathrm{in}}$$

where W and L denote the channel width and channel length of a MOSFET. By selecting (W/L)_n3_ = (W/L)_n2_ and (W/L)_p4_ = (W/L)_p2_. I_out_ can be equal to I_in_. On the other hand, the single-ended transimpedance amplifier stage must also consider two important factors. First, the conversion of cell current into output voltage must be linearly performed. Second, the amplifier voltage output must not saturate within the detectable range of the input current. In order to obtain such conditions, a negative feedback resistor-type transimpedance amplifier is utilized. The utilization of negative feedback transimpedance avoids the non-constant gm effect of the transistor and suppresses the variation of open-loop gain with the input level, yielding higher linearity. The gain of the TIA amplifier can be determined by a resistor value R_f_ expressed as:

(3) $$A_{v}=V_{\mathrm{out}}/I_{\mathrm{out}}=R_{f}$$

In addition, in order to prevent the voltage variation, V_ds_, of transistor M_p4_ due to changing current, the transistor M_p5_ must allow constant current so that voltage V_gs_ across M_p5,_ which is the input terminal of TIA can be maintained at constant level to achieve linear conversion at the output terminal.

Lastly, as for the control amplifier stage, the amplifier controls the cell voltage between WE and RE, and ensures minimum current flows through the reference electrode. Besides, the output terminal of the amplifier is connected to current mirror gate terminal to form negative feedback loop, which controls the voltage at RE through virtual short. Driving the gate terminal of the current mirror offers two advantages. First, the mismatch issue on drain-source voltage of current mirror is prevented [14], which provides more accurate mirror current. Second, instead of the large capacitive value contributed by CE as illustrated in literatures [1] and [4], the parallel combination of capacitors C_gs_ and C_gd_ of the transistor serves as the output load of control amplifier. In this method, the circuit can be made applicable to many sensors without different compensated capacitors. Moreover, it is suggested that the topology of the control amplifier is implemented by a folded cascode amplifier which produces a single pole.

### 2.2. Fully-differential Potentiostat

**Figure 5.**
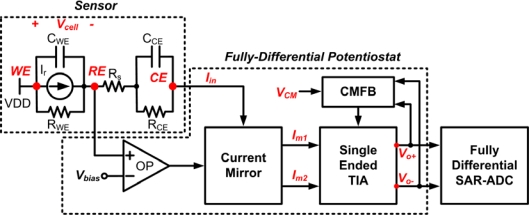
The building blocks of fully-differential potentiostat.

Figure 5 shows the block diagram of a fully-differential potentiostat topology. The function and major components of this topology are analogous to single-ended potentiostat except that output voltage has differential signal mode. The differential mode output voltage signal finds application in fully-differential SAR-ADC [15] for portable system application. The primary advantage of SAR-ADC is low power consumption which is extremely important for portable system. The response time of electrochemical sensor is usually slow and the variation of reacted current may be small, so the low-to-medium operated frequency and medium-to-high resolution properties of SAR-ADC are also suitable for this application.

The fully-differential potentiostat employs a single-to-differential current converter that produces output current, I_m1_ and I_m2_, respectively. The switching process is preceded by converting such current to a differential voltage signal, V_o1_ and V_o2_, which is performed by a fully-differential transimpedance amplifier. Resistors R_f_ are placed in the feedback loop to allow proper conversion of sensor current to voltage signal such that:

(4) $$V_{\mathrm{out}}=V_{o+}-V_{o-}=2\cdot I_{\mathrm{out}}\cdot R_{f}$$

From Equation (4), the output voltage of fully-differential potentiostat doubles the amount of output swing of the readout circuit as compared to a single-ended version. The odd-symmetric I/O characteristic of the differential circuit has the ability to reject second-order harmonic distortion, which in turn increases the linearity of the potentiostat and provides larger detectable current and wider dynamic range. Since the topology involves differential mode operation, the amplifier also necessitates common mode feedback circuit to control the common mode voltage of the output signal. Moreover, the structure of potential control configuration is implemented through a single control amplifier to ensure power consumption issues are minimized.

**Figure 6.**
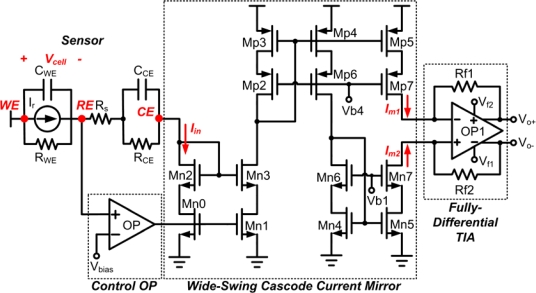
The transistor level schematic of the fully-differential potentiostat.

As shown in Figure 6, the proposed fully-differential potentiostat consists of four blocks to perform the desired operation. This includes wide-swing cascode current mirror, fully-differential transimpedance amplifier, control amplifier, and common mode feedback circuit.

#### a) Wide-Swing Cascode Current Mirror

In the design of single-to-double current mirror of Figure 6, two important conditions are involved to guarantee optimum performance. First, the output signal of control amplifier must be connected to the gate terminal of the mirror transistor, M_n1_, to reduce mismatch of mirror current induced by different drain-source voltage on mirror transistors, M_n2_ and M_n3_. This condition provides precise copying of current to the next stage and allows higher linearity performance to the circuit. Second, because of the fully differential mode, the current mirror must convert the single current from CE to doubled current in the next stage. The conversion is realized through implementing PMOS and NMOS cascode current transistor. However, the accuracy of the mirror current influences the linearity of the potentiostat. To reduce the mismatch between cascade transistors and resolve the detectable current range limitation due to the minimum operational voltage requirement for saturation condition, the wide-swing cascode current mirror is utilized. This enhances output voltage swing while maintaining cascode-type precision and provides higher output impedance.

Furthermore, the symmetrical wide-swing cascode structure is adopted for PMOS and NMOS current mirror simultaneously to reduce the mismatch amount between two reverse currents, I_m1_ and I_m2_. Consequently, the double wide-swing cascode current mirror can provide more accurate current to increase the linearity and provide wider detectable current range of the potentiostat.

#### b) Fully-differential Transimpedance Amplifier

**Figure 7.**
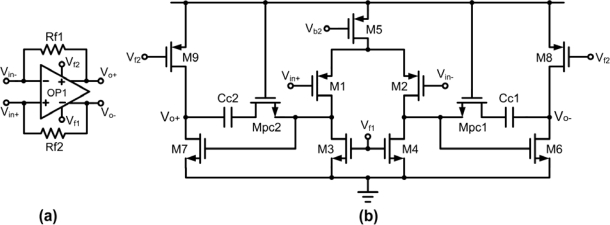
(a) The schematic of FD TIA. (b) The amplifier in FD TIA.

The fully-differential transimpedance amplifier in Figure 6 converts the reverse current into differential voltage signal. Three factors were considered in designing this amplifier. First, the non-constant g_m_ effect from the transistor must be avoided while transimpedance amplifier with negative feedback resistor should be implemented to maintain good linearity of the circuit. Second, the amplifier output range should not saturate within the detectable range of input current. Therefore, as shown in Figure 7, a fully differential two-stage amplifier based topology is utilized in order to attain wider output voltage range capability. In addition, as expressed in Equation (5), the gain has twice the value of a single-ended version:

(5) $$A_{v}=\frac{V_{o+}-V_{o-}}{I_{\mathrm{out}}}=\frac{2\cdot I_{\mathrm{out}}\cdot R_{f}}{I_{\mathrm{out}}}=2\cdot R_{f}$$

**Figure 8.**
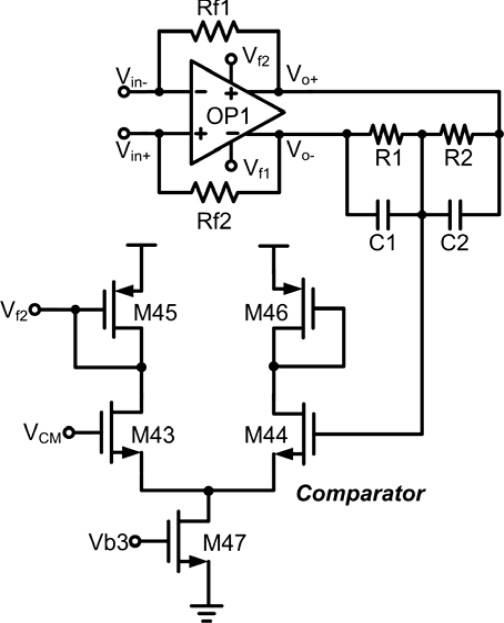
Common mode feedback circuit of the second stage.

Third, a fully-differential amplifier with common mode feedback (CMFB) should be applied to achieve common mode voltage signal. Continuous-time CMFB is chosen in the implementation of the first stage because of the small voltage output swing performance. However, the application of wide sensor redox current causes such performance to become predictably large affecting the input pairs to cut off. Thus to remedy this irregularity, a resistor-type CMFB amplifier, consists of resistors R_1_ and R_2_, is used in the second stage to detect the common-mode voltage of the output signals which in turn compared to the voltage reference, V_ref_, through a comparator as shown in Figure 8. If the common mode feedback circuit is not well compensated, the injection of common-mode signals will cause the amplifier to oscillate or to produce ringing signals. In order to stabilize the differential loop and maintain stability on the amplifier, two capacitors connected in parallel with resistors were utilized.

## 3. Measurement Results and Discussion

**Figure 9.**
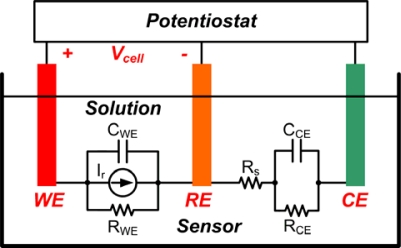
The impedance model of sensor.

The whole course of the experimental analysis is based solely on the equivalent electrochemical model of the sensor built to serve as a platform for all data gathering activity. Figure 9 represents the simple electrical impedance model of an amperometric sensor [14]. In this model, resistor R_WE_ represents the faradaic resistance of WE. WE serves as a surface to which the electrochemical reaction takes place and can be simulated as a current source. Resistors R_CE_ and R_S_, on the other hand, represent the charge transfer resistance at the counter electrode surface and a solution resistance between different electrodes, respectively. Because of the small resistance value of R_S_ it can practically be neglected. In addition, capacitors C_CE_ and C_WE_ are double layer capacitors that existed between the interface of CE and WE and their surrounding electrolyte. Finally, for the simplicity of the equivalent electrochemical model, it can be represented by to two series resistors R_WE_ and R_CE_ at DC.

The experimental environment for testing chip has been set up as shown in Figure 9. Because RE will be biased at constant voltage, V_bias_, the simulated sensor current, I_r_, is generated by varying the voltage signal, V_in_, through a resistor, which is placed between WE and RE such that:

(6) $$I_{r}=\left(V_{\mathrm{in}}-V_{\mathrm{bias}}\right)/R$$

### 3.1. Measurement Results for Single-ended Potentiostat

**Figure 10.**
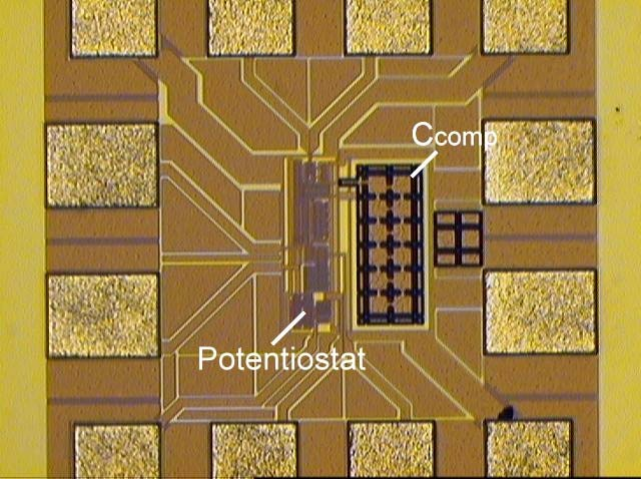
Photomicrograph of single-ended potentiostat.

The prototypes of SE potentiostat and FD potentiostat have been designed and fabricated by TSMC 0.18-μm CMOS technology. Figure 10 shows the photomicrograph of SE potentiostat.

**Figure 11.**
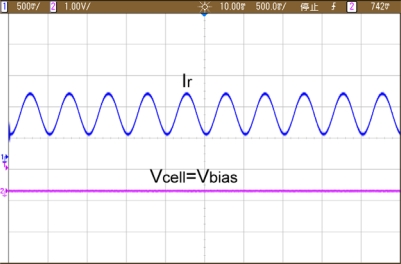
Photomicrograph of single-ended potentiostat.

Figure 11 shows the potentiostat establishes a potential between working and reference electrode. Besides, it forces out the voltage to be the same as V_bias_ by driving or sourcing current from or into counter electrode.

**Figure 12.**
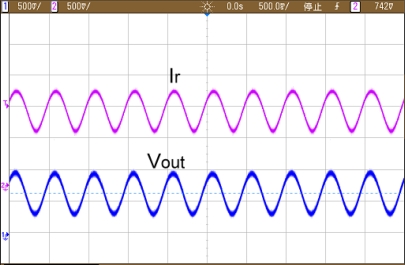
The output voltage with input current.

By varying the input voltage through the resistor, the emulated sensor current, Ir, is swept from 1 μA to 10 μA. The value of the transimpedance gain was chosen to be 100 KΩ. As predicted from the previous analysis, the linear conversion of input current to output voltage can be achieved. This is shown in Figure 12.

### 3.2. Measurement Results for Fully-differential Potentiostat

**Figure 13.**
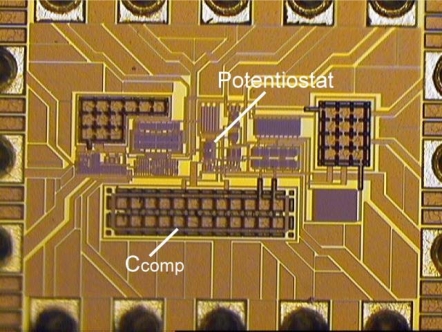
Photomicrograph of fully-differential potentiostat.

Figure 13 shows the photomicrograph of fully-differential potentiostat. It utilizes similar experimental platform environment as Figure 9.

**Figure 14.**
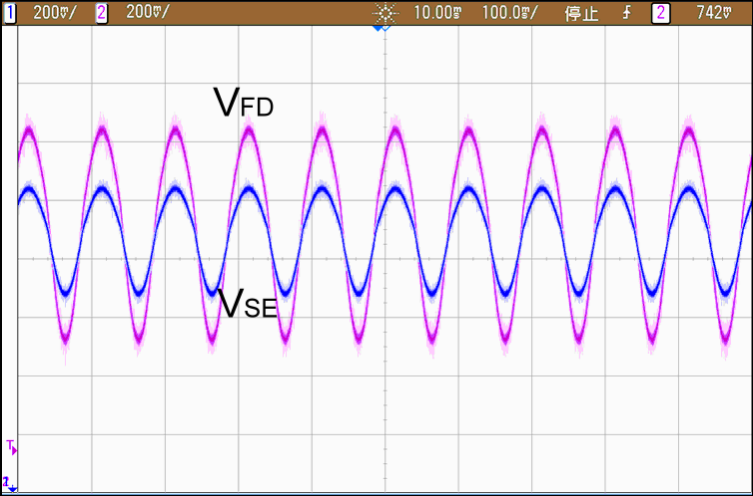
Output swing of SE and FD potentiostat.

In addition, Figure 14 shows the output of the differential output voltage of FD potentiostat. As expected, it has twice the voltage output swing of single-ended potentiostat with the same given transimpedance gain.

The Fast Fourier transformation is utilized to perform the analysis of the circuit linearity. The sine wave current signal is routed through the input and the corresponding output voltage is recorded and analyzed. By recording the fundamental signal power S and the strongest harmonic distortion power, which is introduced due to non-linearity in the conversion D_2_, the spurious-free dynamic range SFDR can be calculated as:

(7) $$\mathrm{SFD}R=10\text{log}\frac{S}{D_{2}}=S(\mathrm{dBm})-D_{2}(\mathrm{dBm})$$

**Figure 15.**
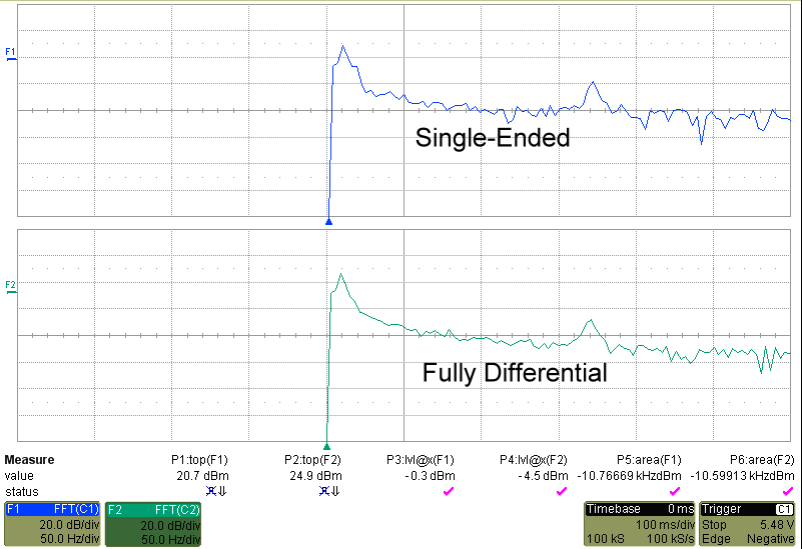
FFT analysis of SE and FD potentiostat.

In Figure 15, the analyzed FFT results of SE and FD potentiostat with the same input current is presented. Because of the differential nature of FD potentiostat, the occurrence of second-order harmonic can be suppressed while the SFDR value is relatively increased as compared to SE.

**Figure 16.**
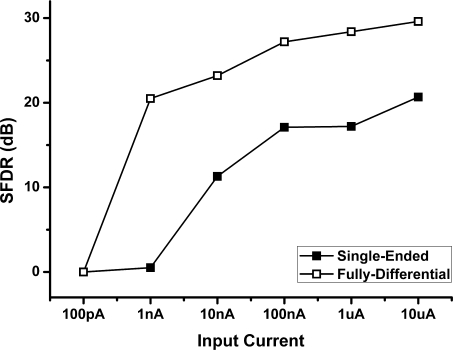
Input current *versus* SFDR of SE and FD poteniotstat.

By varying the amplitude of the input current from 10 uA to 100 pA, the SFDR of the output voltage for FD and SE potentiostat are recorded and plotted in Figure 16. The experimental results show that SE potentiostat measures current from 10 nA to 10 μA with better performance of 10 dB SFDR while FD potentiostat has relatively better linearity in measuring the current from 500 pA to 10 μA. Second, aside from the capability of the FD potentiostat to suppress second-order harmonic distortion, a wide-swing cascade current mirror structure is also adopted to decrease the mismatch amount to provide more accurate current. Thus FD circuit can resolve low-level currents with higher linearity. Additionally, from the definition of dynamic range D.R. = 20log(*I_max_/I_min_*), the FD circuit achieves a wider dynamic range value as compared to SE circuit.

The experimental characteristics of the two proposed potentiostats have been summarized in Table 1.

## 4. Conclusions

In this paper, a single-ended potentiostat topology with a new interface connection between the circuit and electrodes is presented. Such topology can avoid the deviation of cell voltage and linearly convert the cell current to voltage signal. Besides, a fully-differential potentiostat is also presented to alleviate the performance of detectable current and dynamic range from reduced linearity and harmonic distortion, which arises when a low-level sensor current is detected. Moreover, the linearity of the circuit with lesser mismatch amount on the devices is being optimized by employing a wide-swing cascade current mirror. The two proposed potentiostats were implemented using the TSMC 0.18-μm CMOS process. Measurement results show that the performance of FD potentiostat has relatively better linearity, when measuring the current from 500 pA to 10 uA, and a higher dynamic range of 86 dB is achieved. FD potentiostat can also achieve double voltage swing than SE potentiostat. The total power consumption of the entire operation is 307-μW for single-ended design and 1.248-mW for fully-differential with both topologies operating at supply voltage of 1.8V. The core area of the FD potentiostat chip is 0.066 mm^2^ and 0.013 mm^2^ for SE potentiostat. The small die area is suitable for integration with biomedical sensor applications.

## Figures and Tables

**Table 1.** Specification comparison.

	**IEEE IWSOC’05 [14]**	**IEEE ICSSA’95[16]**	**IEEE Sensors J.’09 [17]**	**IEEE ITCAS’09 [18]**	**This Work (SE)**	**This Work (FD)**
**Year**	2005	1995	2009	2009	2009	2009
**Power Supply (V)**	1.8	5	± 0.9V	1.8	1.8	1.8
**Process (um)**	0.18	1	0.18	0.18	0.18	0.18
**I_range_ (A)**	1 n∼200 n	0.1 n∼0.5 u	X	1 n∼1 u	10 n∼10 u	500 p ∼ 10 u
**Dynamic Range (dB)**	46.02	73.98	63	60	60	86
**I_readout_ Electrode**	CE	WE	WE	CE	CE	CE
**Control Part**	SE	SE	FD	SE	SE	SE
**Readout Circuit**	SE	SE	FD	SE	SE	FD
**Output Signal**	Freq.	V_DT_	V_CT_	Freq.	V_CT_	V_CT_
**Core Area (mm^2^)**	0.04	0.6	0.45	0.02	0.013	0.066

*WE: Working electrode; *CE: Counter Electrode; *FD: Fully-differential; *SE : Single-ended *CT: Continuous time *DT: Discrete time; *Freq: frequency output signal
